# Ionic Regulation of T-Cell Function and Anti-Tumour Immunity

**DOI:** 10.3390/ijms222413668

**Published:** 2021-12-20

**Authors:** Pierpaolo Ginefra, Helen Carrasco Hope, Mattia Spagna, Alessandra Zecchillo, Nicola Vannini

**Affiliations:** Department of Oncology, Ludwig Institute for Cancer Research Lausanne, University of Lausanne, 1066 Epalinges, Switzerland; pierpaolo.ginefra@unil.ch (P.G.); mattia.spagna@studenti.unipd.it (M.S.); a.zecchillo3@studenti.uniba.it (A.Z.)

**Keywords:** T cell, ions, tumour microenvironment, immunomodulation, nutrient competition

## Abstract

The capacity of T cells to identify and kill cancer cells has become a central pillar of immune-based cancer therapies. However, T cells are characterized by a dysfunctional state in most tumours. A major obstacle for proper T-cell function is the metabolic constraints posed by the tumour microenvironment (TME). In the TME, T cells compete with cancer cells for macronutrients (sugar, proteins, and lipid) and micronutrients (vitamins and minerals/ions). While the role of macronutrients in T-cell activation and function is well characterized, the contribution of micronutrients and especially ions in anti-tumour T-cell activities is still under investigation. Notably, ions are important for most of the signalling pathways regulating T-cell anti-tumour function. In this review, we discuss the role of six biologically relevant ions in T-cell function and in anti-tumour immunity, elucidating potential strategies to adopt to improve immunotherapy via modulation of ion metabolism.

## 1. Introduction

T lymphocytes undergo a metabolic reprogramming upon TCR-stimulation, which sustains the biosynthetic requirements of clonal expansion and differentiation. Indeed, the engagement of specific metabolic pathways requires the presence of particular metabolites that are not only necessary to promote the synthesis of ATP and macromolecules but also to mediate signalling regulation of T-cell function and fate [1,2,3]. The role of metabolism in modulating T-cell responses becomes evident in the context of anti-tumour immunity, where cancer cells acquire suppressive mechanisms to evade the immune system [4]. Nutrient competition between cancer and immune cells in the tumour microenvironment (TME) or the secretion of cancer-cell suppressive metabolic waste products (e.g., adenosine or kynurenine) have been deeply studied during the last decade [5,6], and a myriad of promising interventions has been developed to overcome these metabolic barriers and to boost anti-tumour T-cell responses [7,8].

The role of glucose, amino acids, and lipids in the regulation of T-cell responses against cancer has been studied and reviewed extensively elsewhere [9,10,11,12,13,14]. Here, we focus on the underrepresented function of ions. T cells require an appropriate balance of extracellular and intracellular ion levels to maintain cell and mitochondrial membrane potential (∆Ψm). Furthermore, ions operate as second messengers for TCR signalling, act as cofactors for a multitude of enzymes, and interact with DNA to stabilise its structure. Disturbances in ionic concentrations or in the expression of ionic channels are detrimental for T-cell performance and lead to the appearance of immune-related diseases. Although it is well-known that ionic homeostasis is essential for T-cell survival and activity, the functional relevance of ions within tumours remains poorly understood. Recent reports have shown that tumour necrotic cells release ions within the TME and that several cancer types modify ion-channel expression to adapt to the ionic conditions of the TME [15,16]. In this review, we will discuss how ions shape T-cell immunity and describe the latest advances in the context of anti-tumour immunity. Specifically, we will focus on six ions with potential translational application: potassium, manganese, zinc, selenium, iron, and magnesium.

## 2. Potassium

**Figure 1:**
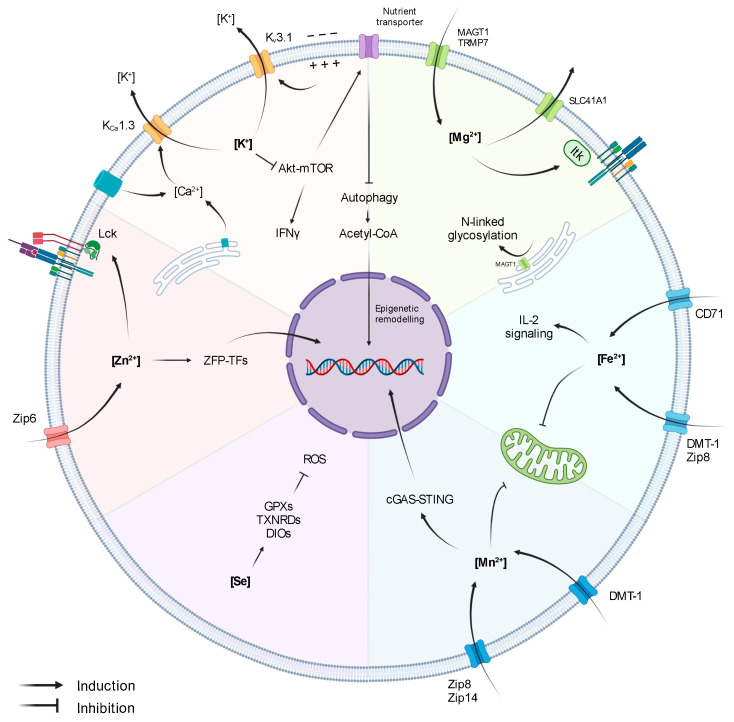
Influence of Ions on T-cell activity.

Potassium (K^+^) is the most abundant ion in mammalian cells, with intracellular K^+^ levels ([K^+^]_i_) reaching ~130 mM, while extracellular levels [K^+^]_e_ are ~3–5 mM [17,18]. In T lymphocytes, K^+^ gradient is balanced through the action of two ion channels mediating K^+^ efflux: the voltage-gated K_v_1.3 and the Ca^2+^-activated K_Ca_3.1 channels (Figure 1) [19]. Alterations in the expression of these channels and, subsequently, in [K^+^]_i,_ lead to aberrant T-cell functionality.

The role of K^+^ in T cells is tightly linked to Ca^2+^ signalling. Upon antigen recognition, the activation of TCR signalling triggers the opening of Ca^2+^ channels (Orai1 in the ER and CRAC channels in the cell membrane), leading to increased intracellular Ca^2+^ levels. Importantly, Ca^2+^ induces NFAT expression and subsequent IL-2 production and T-cell activation [20]. However, the first wave of Ca^2+^ release generates an electrochemical imbalance that depolarises the membrane and hampers further Ca^2+^ influx. Membrane depolarisation and the elevated Ca^2+^ levels activate K_v_1.3 and K_Ca_3.1, respectively, promoting K^+^ efflux, thus restoring membrane potential and enabling continuous Ca^2+^ entry and signalling amplification (Figure 1) [19,21]. Indeed, a blockade of K_v_1.3 and K_Ca_3.1 reduces Ca^2+^ signalling, demonstrating the key role of K^+^ gradient in preserving the equilibrium of the membrane potential upon TCR stimulation and ensuring efficient T-cell activation [22]. In accordance, K_v_1.3 and K_Ca_3.1 are highly expressed upon T-cell activation, and they co-localize at the immunological synapse, together with CRAC channels [15,21,23]. Moreover, K_v_1.3 and K_Ca_3.1 have also been shown to influence T-cell migratory capacity [24,25].

Importantly, K_v_1.3 and K_Ca_3.1 expression vary between T-cell subsets. It has been described that T_h_1 and T_h_2 cells predominantly express K_Ca_3.1, whilst T_h_17 and T_regs_ express K_v_1.3. In fact, K_Ca_3.1^−/−^ mice are resistant to the induction of autoimmune colitis, characterised by the presence of autoreactive T_h_1 cells. In this model, depletion of K_Ca_3.1 disrupted T_h_1 activity without affecting the functionality of T_regs_ and T_h_17 cells [26]. Similarly in humans, effector-memory T cells (CD45RA^−^CCR7^+^; T_em_) are highly dependent on K_v_1.3 for Ca^2+^ signalling, whereas central-memory T cells (CD45RA^−^CCR7^−^; T_cm_) are mostly dependent on K_Ca_3.1, and K_v_1.3 inhibition only mildly affects their functionality [27,28]. The differences in expression levels are interesting from an immunotherapeutic perspective, as the application of K^+^ channel blockers could be used to target specific T-cell populations.

In the context of anti-tumour T-cell responses, it has been shown that necrotic cancer cells within hypoxic areas release large amounts of K^+^, which directly inhibit effector functions of murine and human CD8^+^ T cells [15,18]. Mechanistically, T-cell suppression derived from exposure to high extracellular [K^+^] ([K^+^]_e_) is not directly caused by membrane-potential variations or Ca^2+^ signalling alterations but is rather due to an increase in intracellular [K^+^]_i_, which affects the Akt-mTOR pathway (Figure 1) [15,18]. In addition, it has been described that hypoxia downregulates K_v_1.3 and K_Ca_3.1 [29], suggesting that intracellular [K^+^]_I_ could be further augmented in the TME through other synergistic mechanisms. Importantly, overexpression of K_Ca_3.1 decreases intracellular [K^+^]_I_ and restores T-cell Akt-mTOR signalling and IFNγ secretion, resulting in improved tumour growth control and survival [15]. These reports indicate that levels of [K^+^]_I_ and K^+^ channels in T cells might be used as markers of T-cell fitness within tumours. Accordingly, K_v_1.3 and K_Ca_3.1 activity in CD8^+^ T cells derived from head- and neck-cancer patients correlate with increased T-cell infiltration and functionality [25,30,31]. Moreover, K^+^ is also an important cofactor for the glycolytic enzyme hexokinase-II (HK-II), suggesting that K^+^ might not only be involved in the regulation of anti-tumour immunity but also in the adaptation of cancer-cell metabolism in the TME. Altogether, these studies support the concept that K^+^ acts as a suppressive element of anti-tumour immunity. However, a more recent report by Vodnala et al. (2019) showed that despite dampening T-cell effector functions, mTOR inactivation derived from high [K^+^]_e_ is accompanied by a decreased nutrient uptake, which initiates a starvation response. The authors define this state as ‘functional caloric restriction’, characterised by autophagy induction and acetyl-CoA-dependent epigenetic remodelling (Figure 1). Specifically, exposure to [K^+^]_e_ reduced the acetylation of effector/exhaustion-associated loci of genes such as *Pdcd1* (PD1), *Cd244* (2B4), and *Havcr2* (Tim-3) while preserving T-cell stemness through the induction of TCF1 expression. Consequently, T cells exposed to high [K^+^]_e_ during in vitro expansion enhanced T-cell persistence and anti-tumour response upon adoptive cell transfer in a B16 melanoma mouse model [32]. On the contrary, CD19-directed human CAR-T cells cultured for 48 h in cerebrospinal fluid (CSF), which contains low concentrations of glucose and K^+^, expressed elevated levels of genes encoding for survival and memory markers (e.g., BCL2, IL7R) and lower levels of effector genes (e.g., IFNγ, GrB, Tbet) [33]. Although plenty of evidence points at K^+^ as an interesting target for immunotherapy, the dual roles of K^+^ in anti-tumour T cells, the discrepancies observed in murine and human settings, and the direct effect of K^+^ on cancer cells indicate that further investigations are required to unveil the best strategy to exploit K^+^ in cancer therapy.

## 3. Manganese

Manganese (Mn^2+^) is one of the most abundant metals found in the tissues of mammals, and it is crucial for intracellular processes regulating energy production, development, antioxidant defence and immune response [34]. Indeed, uptake, retention, and excretion of Mn^2+^ are tightly regulated due to its key role as cofactor of a variety of enzymes, such as Mn^2+^ superoxide dismutase (SOD), glutamine synthetase (GS), arginase, and pyruvate carboxylase. Intracellular Mn^2+^ homeostasis is regulated through non-exclusive metal-ion transporters, including divalent metal transporter A (DMT1), calcium channel-dependent protein, and metal-transporter-family proteins like Zip8 and Zip14 (Figure 1) [35,36,37].

Mn^2+^ is present in all compartments. However, most intracellular Mn^2+^ is stored in the Golgi apparatus and in the mitochondria [38]. When supplemented at high concentrations in culture media, Mn^2+^ accumulates in the mitochondria and in the nucleus, impairing mitochondrial activity and inducing DNA damage (Figure 1) [39]. In HeLa and in THP1 cells, Mn^2+^ release from the mitochondria and Golgi to the cytosol increases the sensitivity of the DNA sensor cGAS and the downstream adaptor protein STING, which, in turn, induces type I IFNs and cytokine production [40]. However, its function in both adaptive and innate immunity has been poorly investigated. A recent study has shown that Mn^2+^ supplementation improved tumour-specific antigen presentation acting on macrophages and dendritic cell maturation [41]. As a consequence, both dendritic cells and macrophage maturation contribute to CD8^+^ T-cell activation and better tumour control in a B16 melanoma model. Congruently, as first reported in the 1980s, Mn^2+^ supplementation leads to a significant increase in the number of TILs [41,42,43]. In addition, Mn^2+^ treatment increases cytokine production capacity in both CD8^+^ T and NK infiltrating tumours, while depletion of Mn^2+^ from the diet results in a reduced T cells differentiation and increased tumour size. Mn^2+^ anti-tumoural activities, such as increased TIL number, function, or shifting macrophage polarization to a more anti-tumoural phenotype, has been exploited in combination with conventional chemotherapy and immune checkpoint blockade therapy to boost anti-tumour response [44,45]. Indeed, Mn^2+^ can induce type I IFN production and dendritic cell maturation, similarly to STING agonist, making Mn^2+^ a potential novel adjuvant for cancer vaccines (Figure 1) [41,46]. Taken together, due to its promiscuous effect in stimulating both myeloid (dendritic cells) and lymphoid (CD8^+^ T cells and NK) compartments, Mn^2+^ metabolism emerges as a potential novel target for anti-tumour therapies.

## 4. Zinc

Zinc (Zn^2+^) is the second most abundant trace metal in the human body after iron. It is an essential component of several proteins [47] and participates in a variety of cellular processes, including cell proliferation, differentiation, redox regulation, and apoptosis. [48,49,50]. Zn^2+^ is mostly intracellular and conjugated to zinc-binding proteins [51]. Zn^2+^ homeostasis is tightly controlled by a variety of transporters and chaperone proteins called metallothioneins [52]. Importantly, Zn^2+^ regulates both innate and adaptive immunity [53,54]. Chronic Zn^2+^ deficiency impairs proper T-cell development, differentiation, and function [55]. Indeed, Zn^2+^ deficiency reduces expression of the cytotoxic T lymphocyte marker CD73 in patients with sickle cell anaemia [56] and leads to a significant reduction of thymus-derived hormone thymulin, regulating T-cell differentiation and maturation [57]. Zn^2+^ is also involved in T-cell activation and differentiation, being involved in the interaction between the short cytoplasmatic domain of CD4 or CD8α with p56^lck^ (Figure 1) [58]. Upon TCR signalling, cytoplasmatic Zn^2+^ concentration increases within 1 min due to the rapid upregulation of the zinc transporter Zip6 (Figure 1) [59], leading to Zap70 phosphorylation and sustained calcium influx, which supports T-cell proliferation in suboptimal conditions [59]. Moreover, inhibition of Zn^2+^ influx through Zip6 silencing impairs T-cell activation, resulting in reduced expression of activation markers, such as CD25 and CD69, and reduced production of cytokines, such as IL-2 [60]. Similarly, Zn^2+^ depletion blocks the ERK1/2 and PI3K/Akt pathways, inhibiting T-cell activation [61,62]. While the direct effect of Zn^2+^ on tumour growth has not been elucidated yet, few studies have indicated a potential immunosuppressive role of Zn^2+^ both in vitro [63] and in the tumour microenvironment [64]. Notably, it has been shown that a Zn^2+^-rich diet can promote prostate carcinogenesis and increase the risk of prostate cancer progression [65].

Finally, in the B16F10 murine melanoma model, it has been observed that TILs upregulate metallothieins and zinc-finger transcription factors, such as GATA-3 and IKZF2 (Figure 1) [66], indicating a possible role of Zn^2+^ homeostasis in T-cell differentiation and exhaustion within the TME. The evidence gathered so far places Zn^2+^ metabolism as a potential target to dampen the immunosuppressive mechanism adopted by cancer cells. However, how Zn^2+^ acts as an immunosuppressive factor and which zinc-dependent proteins are involved in the process has yet to be defined.

## 5. Selenium

Selenium (Se^2−^) is taken up through the diet in either organic forms, seleno-L-methionine (SeMet) and seleno-L-cysteine (SeCys), or as inorganic forms, selenide and selenite, which are all ultimately metabolized within mammalian cells into SeCys. Indeed, SeCys, also known as the 21st amino acid, is an essential element of selenoprotein catalytic sites [67,68]. In humans, 25 genes encoding for selenoproteins have been identified, with most of them involved in the regulation of redox balance and protection against oxidative stress. Enzymatic glutathione peroxidases (GPXs), thioredoxin reductases (TXNRDs), or iodothyronine deiodinases (DIOs) (Figure 1), as well as the non-enzymatic selenoprotein P (SELENOP) and selenoprotein K (SELENOK), are amongst the most important selenoproteins [67,68]. SELENOP is known to be one of the most important Se^2−^ carriers in circulation. On the other hand, the molecular mechanisms involved in Se^2−^ cellular uptake have not yet been completely elucidated [67,68].

In an immunological context, Se^2−^ supplementation boosts immune function via regulation of selenoprotein levels. Shrimali et al. (2008) generated mice with T-cell-specific ablation of the SeCys tRNA^[Ser]Sec^ and described that loss of selenoprotein synthesis in T cells leads to ROS hyperproduction and suppression of T-cell expansion after TCR stimulation [69]. Furthermore, another report by Verma et al. (2011) showed that T cells lacking SELENOK, an ER transmembrane protein that regulates Ca^2+^ flux, display reduced Ca^2+^ signalling during T-cell activation and, subsequently, defective immune responses during viral infection [70]. These investigations, together with epidemiological studies showing that Se-deficient diets are associated with a loss of immunocompetence [71], indicate that Se^2−^ levels and selenoproteins are essential for appropriate regulation of T-cell-mediated immunity.

Even though the role of Se^2−^ in T-cell anti-tumour responses has been poorly elucidated, a combination of preclinical and clinical studies indicate that increased Se^2−^ serum levels are associated with overall improved survival in patients [72]. In particular, sodium-selenite-enriched diets have shown to reduce tumour size in mice by enhancing the cytotoxicity of both CD8^+^ T cells and NK cells, suggesting a direct effect on anti-tumour immunity [73]. Importantly, selenoprotein GPX4 has been described as a fate and functional determinant of TILs. Specifically, decreased GPX4 expression in TILs is associated with an accumulation of oxidized lipids that induces T-cell death via ferroptosis [74]. GPX4-mediated regulation of ferroptosis is also a survival mechanisms of cancer cells, which can increase GPX4 levels through the induction of selenophosphate synthetase 2 (SEPHS2), an enzyme involved in SeCys biosynthesis [75]. Altogether, these reports indicate that cancer progression is influenced by Se^2−^ levels, by both affecting cancer-cell survival and immune-cell function, opening the way to Se^2−^ modulation as a possible future strategy to boost cancer immunotherapy.

## 6. Magnesium

Magnesium (Mg^2+^) is the most abundant divalent cation in eukaryotic cells (~10–30 mM). While only ~5% of intracellular Mg^2+^ is found free ([Mg^2+^]_i_), most of it is complexed to ATP or bonded to other molecules functioning as a cofactor. In T cells, [Mg^2+^]_i_ levels are finely regulated by the ion channels MAGT1, TRPM7, mediating Mg^2+^ influx, and SLC41A1, mediating Mg^2+^ efflux through Na^+^ exchange (Figure 1) [76].

T-cell antigen recognition is followed by a rapid transient Mg^2+^ influx, which acts as second messenger in TCR signalling [77,78]. Specifically, Mg^2+^ directly interacts with IL-2-inducible T-cell kinase (ITK) promoting its activation (Figure 1) [79]. On the contrary, lymphocyte activation in low [Mg^2+^] conditions limits CD69 and CD25 upregulation, Ca^2+^ influx, and cell proliferation [79]. Indeed, mice fed Mg^2+^-restricted diets and infected with influenza A virus have reduced numbers of virus-specific T cells [79]. Furthermore, patients carrying loss-of-function mutations in *MAGT1* gene develop a rare primary immunodeficiency known as XMEN disease (‘X-linked immunodeficiency with Mg^2+^ defect, Epstein-Barr virus (EBV) infection, and neoplasia’). T cells from patients with XMEN disease exhibit limited Mg^2+^ influx and recapitulate most of the features observed in low [Mg^2+^] conditions (i.e., deficient TCR signalling, Ca^2+^ influx, T-cell activation and proliferation) [77,78,80]. Interestingly, MAGT1 localizes in the ER, where it mediates N-linked glycosylation, a post-translational modification influencing protein half-life (Figure 1) [81,82]. In XMEN patients, CD8^+^ T cells lose CD70 and NKG2D expression due to its diminished glycosylation, which has been linked to an increased susceptibility to EBV infection [81,82,83].

Mg^2+^ is important for the stabilisation of DNA structure and operates as a cofactor for enzymes involved in DNA repair, suggesting that Mg^2+^ deprivation might lead to accumulation of DNA damage and carcinogenesis. Accordingly, low Mg^2+^ intake is associated with higher risk of pancreatic, lung, and breast cancer [84,85,86], while alterations in MAGT1 and SLC41A1 expression have been associated with aggressive colorectal cancers and pancreatic ductal adenocarcinomas (PDAC) [87,88]. Interestingly, Diao et al. (2017) described that chronically activated CD8^+^ T cells in hepatitis B virus (HBV)-infected patients show a decline in [Mg^2+^]_i_ and MAGT1 expression associated with PD-1 upregulation and loss of NKG2D [89]. To date, this phenotype has not been identified in the exhausted TILs. However, the necrosis-derived release of ions [15] added to the alterations in the expression of Mg^2+^ transporters in cancer cells suggests that Mg^2+^ levels might vary in the TME and thus have an immunomodulatory role within the TME.

## 7. Iron

Iron (Fe^2+^) is an essential element involved in several enzymatic reactions and cellular processes, such as proliferation, DNA synthesis, metabolism [90], and immune function [91,92]. For this reason, Fe^2+^ levels are tightly regulated. Most of the Fe^2+^ delivered to the cells is bound to transferrin protein (Tf). The Tf-iron complex is taken up by the cells through transferrin receptor (CD71) endocytosis. Notably, T cells can also take up Fe^2+^ via non-specific metal-ion transporters, like DMT-1 and ZIP-8 (Figure 1) [93,94]. During activation, T cells increase expression of CD71 (Figure 1) [95]. On the contrary, anergic T cells have reduced expression of CD71 [96]. Reduced Fe^2+^ uptake due to defective Tf-receptor endocytosis impairs T-cell function and results in severe immunodeficiency [97]. Furthermore, reduced intracellular Fe^2+^ levels impaired CD25 expression and IL-2R signalling and compromised mitochondrial function in T cells (Figure 1). Notably, Fe^2+^ supplementation in an iron-deficiency culture system restore proper mitochondrial potential and biogenesis [98].

A recent report revealed a role of Fe^2+^ in an inflammatory context. In autoinflammatory diseases, iron deposition is frequently observed. According to Wang et al., Fe^2+^ promotes proinflammatory cytokine production in immune cells, including T cells [99]. On the other hand, it has been reported that Fe^2+^ is released by tumour-associated macrophages (TAMs) and tumour-associated neutrophils (TANs) in the TME. In this scenario, Fe^2+^ might sustain TAMs and TANs in supporting cancer progression and impairing T and B cell activity by inducing cell death. Fe^2+^ has been involved in the induction of ferroptosis by mechanisms that are still poorly understood [100]. Although the impact of Fe^2+^ secretion by TAMs and TANs is not directly proven, it is likely that high Fe^2+^ levels may contribute to the induction of ferroptosis in T cells and cancer cells. Furthermore, TAMs and TANs can also impair proper APC maturation and antigen presentation [101,102]. In light of the cited reports, altering Fe^2+^ concentration in the tumour microenvironment could be a promising approach to improve current therapies [102,103].

## 8. Conclusions and Perspectives

**Figure 2:**
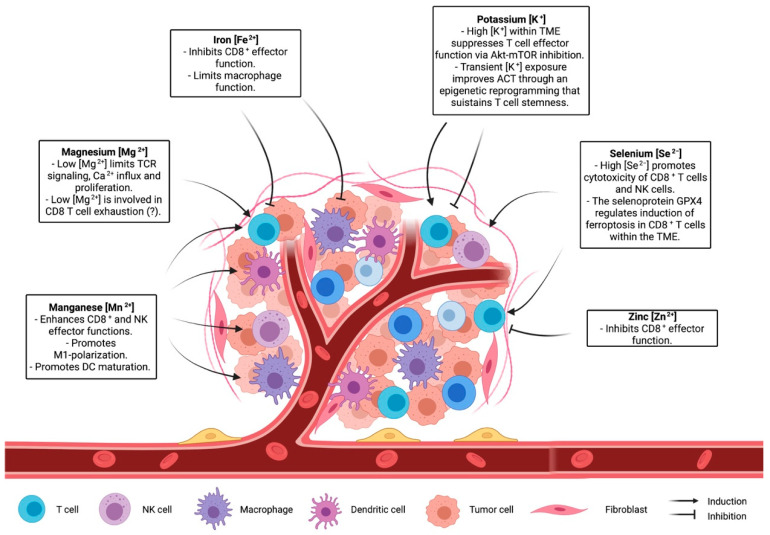
The role of ions in shaping the immune landscape of the microenvironment.

Many clinical trials have demonstrated therapeutic efficacy of T-cell based immunotherapy, which exploits the capacity of T cells to recognize and kill a specific target, including cancer cells [104]. While it is well established that metal ions can regulate immune-system and T-cell function and metabolism, it is not clear how the manipulation of ion concentrations in the TME can improve T-cell activity and possibly T-cell-based immunotherapy. Recent studies cited in this review underline the role of ions in shaping T-cell capacity controlling tumour growth (Figure 2). Although there is an increased interest in understanding the role of ions in the context of the tumour microenvironment [15,105], the complex interplay between ion concentration, immune cells, and cancer cells has not been sufficiently investigated. Recently, cutting-edge gene-targeting technologies, like CRISPR, have been adopted to reveal processes involved in nutrient sensing and consumption in T cells in vivo [106]. Implementing these approaches to ion channels and ion-dependent enzymes would provide a deeper view on the molecular processes orchestrated by specific ions and on how these processes influence T-cell activity. Another challenging aspect is the development of strategies capable of locally altering ionic concentration in the TME. While adequate diet and nutrient supplementation can modulate ion blood levels, it is not known whether a systemic change in ion intake might lead to a local effect. Further studies are needed to elucidate whether a tailored supplementation of a given ion would be adequate to optimize immune function in the TME. Another possibility would be to design methods to locally deliver or deplete a specific ion. Canale et al. used engineered bacteria to locally deliver arginine in the TME, enabling metabolic modulation of the tumour microenvironment and improving adaptive immune responses against cancer cells [107]. A similar approach suited for ions would provide a tool to alter ion concentration only in the TME. Indeed, a large body of evidence has shown how metabolism and nutrient consumption are key factors for a proper and robust anti-tumour immune response [12,108]. In this context, elucidating the role of ions in both homeostatic and anti-tumour T-cell activity might help in the development of novel strategies aimed to improve T-cell-based therapies.

## References

[1] Buck M.D., O’Sullivan D., Pearce E.L. (2015). T cell metabolism drives immunity. J. Exp. Med..

[2] Buck M.D., Sowell R.T., Kaech S.M., Pearce E.L. (2017). Metabolic Instruction of Immunity. Cell.

[3] Geltink R.I.K., Kyle R.L., Pearce E.L. (2018). Unraveling the Complex Interplay between T Cell Metabolism and Function. Annu. Rev. Immunol..

[4] Anderson K.G., Stromnes I.M., Greenberg P.D. (2017). Obstacles Posed by the Tumor Microenvironment to T cell Activity: A Case for Synergistic Therapies. Cancer Cell.

[5] Sugiura A., Rathmell J.C. (2018). Metabolic Barriers to T-cell function in Tumors. J. Immunol..

[6] DePeaux K., Delgoffe G.M. (2021). Metabolic barriers to cancer immunotherapy. Nat. Rev. Immunol..

[7] Hope H.C., Salmond R.J. (2019). Targeting the tumor microenvironment and T cell metabolism for effective cancer immunotherapy. Eur. J. Immunol..

[8] O’Sullivan D., Sanin D.E., Pearce E.J., Pearce E.L. (2019). Metabolic interventions in the immune response to cancer. Nat. Rev. Immunol..

[9] Hope H.C., Salmond R.J. (2021). The Role of Non-essential Amino Acids in T Cell Function and Anti-tumour Immunity. Arch. Immunol. Ther. Exp..

[10] Reinfeld B.I., Rathmell W.K., Kim T.K., Rathmell J.C. (2021). The therapeutic implications of immunosuppressive tumor aerobic glycolysis. Cell Mol. Immunol..

[11] Kelly B., Pearce E.L. (2020). Amino Assets: How Amino Acids Support Immunity. Cell Metab..

[12] Li X., Wenes M., Romero P., Huang S.C., Fendt S.M., Ho P.C. (2019). Navigating metabolic pathways to enhance antitumour immunity and immunotherapy. Nat. Rev. Clin. Oncol..

[13] Lukey M.J., Katt W.P., Cerione R.A. (2017). Targeting amino acid metabolism for cancer therapy. Drug Discov. Today.

[14] Yin Z., Bai L., Li W., Zeng T., Tian H., Cui J. (2019). Targeting T cell metabolism in the tumor microenvironment: An anti-cancer therapeutic strategy. J. Exp. Clin. Cancer Res..

[15] Eil R., Vodnala S.K., Clever D., Klebanoff C.A., Sukumar M., Pan J.H., Palmer D.C., Gros A., Yamamoto T.N., Patel S.J. (2016). Ionic immune suppression within the tumour microenvironment limits T cell effector function. Nature.

[16] Litan A., Langhans S.A. (2015). Cancer as a channelopathy: Ion channels and pumps in tumor development and progression. Front. Cell. Neurosci..

[17] Gurusamy D., Clever D., Eil R., Restifo N.P. (2017). Novel “Elements” of Immune Suppression within the Tumor Microenvironment. Cancer Immunol. Res..

[18] Ong S.T., Ng A.S., Ng X.R., Zhuang Z., Wong B.H.S., Prasannan P., Kok Y.J., Bi X., Shim H., Wulff H. (2019). Extracellular K(+) Dampens T Cell Functions: Implications for Immune Suppression in the Tumor Microenvironment. Bioelectricity.

[19] Feske S., Wulff H., Skolnik E.Y. (2015). Ion channels in innate and adaptive immunity. Annu. Rev. Immunol..

[20] Hwang J.-R., Byeon Y., Kim D., Park S.-G. (2020). Recent insights of T cell receptor-mediated signaling pathways for T cell activation and development. Exp. Mol. Med..

[21] Cahalan M.D., Chandy K.G. (2009). The functional network of ion channels in T lymphocytes. Immunol. Rev..

[22] Beeton C., Wulff H., Standifer N.E., Azam P., Mullen K.M., Pennington M.W., Kolski-Andreaco A., Wei E., Grino A., Counts D.R. (2006). Kv1.3 channels are a therapeutic target for T cell-mediated autoimmune diseases. Proc. Natl. Acad. Sci. USA.

[23] Panyi G., Vámosi G., Bacsó Z., Bagdány M., Bodnár A., Varga Z., Gáspár R., Mátyus L., Damjanovich S. (2004). Kv1.3 potassium channels are localized in the immunological synapse formed between cytotoxic and target cells. Proc. Natl. Acad. Sci. USA.

[24] Sim J.H., Kim K.S., Park H., Kim K.J., Lin H., Kim T.J., Shin H.M., Kim G., Lee D.S., Park C.W. (2017). Differentially Expressed Potassium Channels Are Associated with Function of Human Effector Memory CD8(+) T Cells. Front. Immunol..

[25] Chimote A.A., Balajthy A., Arnold M.J., Newton H.S., Hajdu P., Qualtieri J., Wise-Draper T., Conforti L. (2018). A defect in KCa3.1 channel activity limits the ability of CD8(+) T cells from cancer patients to infiltrate an adenosine-rich microenvironment. Sci. Signal..

[26] Di L., Srivastava S., Zhdanova O., Ding Y., Li Z., Wulff H., Lafaille M., Skolnik E.Y. (2010). Inhibition of the K+ channel KCa3.1 ameliorates T cell-mediated colitis. Proc. Natl. Acad. Sci. USA.

[27] Hu L., Wang T., Gocke A.R., Nath A., Zhang H., Margolick J.B., Whartenby K.A., Calabresi P.A. (2013). Blockade of Kv1.3 potassium channels inhibits differentiation and granzyme B secretion of human CD8+ T effector memory lymphocytes. PLoS ONE.

[28] Panyi G., Beeton C., Felipe A. (2014). Ion channels and anti-cancer immunity. Philos. Trans. R. Soc. Lond. B Biol. Sci..

[29] Conforti L., Petrovic M., Mohammad D., Lee S., Ma Q., Barone S., Filipovich A.H. (2003). Hypoxia regulates expression and activity of Kv1.3 channels in T lymphocytes: A possible role in T cell proliferation. J. Immunol..

[30] Chimote A.A., Hajdu P., Sfyris A.M., Gleich B.N., Wise-Draper T., Casper K.A., Conforti L. (2017). Kv1.3 Channels Mark Functionally Competent CD8+ Tumor-Infiltrating Lymphocytes in Head and Neck Cancer. Cancer Res..

[31] Newton H.S., Gawali V.S., Chimote A.A., Lehn M.A., Palackdharry S.M., Hinrichs B.H., Jandarov R., Hildeman D., Janssen E.M., Wise-Draper T.M. (2020). PD1 blockade enhances K(+) channel activity, Ca(2+) signaling, and migratory ability in cytotoxic T lymphocytes of patients with head and neck cancer. J. Immunother. Cancer.

[32] Vodnala S.K., Eil R., Kishton R.J., Sukumar M., Yamamoto T.N., Ha N.H., Lee P.H., Shin M., Patel S.J., Yu Z. (2019). T cell stemness and dysfunction in tumors are triggered by a common mechanism. Science.

[33] Wang X., Huynh C., Urak R., Weng L., Walter M., Lim L., Vyas V., Chang W.C., Aguilar B., Brito A. (2021). The Cerebroventricular Environment Modifies CAR T Cells for Potent Activity against Both Central Nervous System and Systemic Lymphoma. Cancer Immunol. Res..

[34] Chen P., Bornhorst J., Aschner M. (2018). Manganese metabolism in humans. Front. Biosci..

[35] Crossgrove J.S., Yokel R.A. (2004). Manganese distribution across the blood-brain barrier III. The divalent metal transporter-1 is not the major mechanism mediating brain manganese uptake. Neurotoxicology.

[36] Liuzzi J.P., Aydemir F., Nam H., Knutson M.D., Cousins R.J. (2006). Zip14 (Slc39a14) mediates non-transferrin-bound iron uptake into cells. Proc. Natl. Acad. Sci. USA.

[37] Horning K.J., Caito S.W., Tipps K.G., Bowman A.B., Aschner M. (2015). Manganese Is Essential for Neuronal Health. Annu. Rev. Nutr..

[38] Carmona A., Roudeau S., Perrin L., Veronesi G., Ortega R. (2014). Environmental manganese compounds accumulate as Mn(II) within the Golgi apparatus of dopamine cells: Relationship between speciation, subcellular distribution, and cytotoxicity. Metallomics.

[39] Morello M., Canini A., Mattioli P., Sorge R.P., Alimonti A., Bocca B., Forte G., Martorana A., Bernardi G., Sancesario G. (2008). Sub-cellular localization of manganese in the basal ganglia of normal and manganese-treated rats An electron spectroscopy imaging and electron energy-loss spectroscopy study. Neurotoxicology.

[40] Wang C., Guan Y., Lv M., Zhang R., Guo Z., Wei X., Du X., Yang J., Li T., Wan Y. (2018). Manganese Increases the Sensitivity of the cGAS-STING Pathway for Double-Stranded DNA and Is Required for the Host Defense against DNA Viruses. Immunity.

[41] Lv M., Chen M., Zhang R., Zhang W., Wang C., Zhang Y., Wei X., Guan Y., Liu J., Feng K. (2020). Manganese is critical for antitumor immune responses via cGAS-STING and improves the efficacy of clinical immunotherapy. Cell Res..

[42] Rogers R.R., Garner R.J., Riddle M.M., Luebke R.W., Smialowicz R.J. (1983). Augmentation of murine natural killer cell activity by manganese chloride. Toxicol. Appl. Pharmacol..

[43] Smialowicz R.J., Rogers R.R., Riddle M.M., Luebke R.W., Rowe D.G., Garner R.J. (1984). Manganese chloride enhances murine cell-mediated cytotoxicity: Effects on natural killer cells. J. Immunopharmacol..

[44] Yang G., Xu L., Chao Y., Xu J., Sun X., Wu Y., Peng R., Liu Z. (2017). Hollow MnO2 as a tumor-microenvironment-responsive biodegradable nano-platform for combination therapy favoring antitumor immune responses. Nat. Commun..

[45] Song M., Liu T., Shi C., Zhang X., Chen X. (2016). Bioconjugated Manganese Dioxide Nanoparticles Enhance Chemotherapy Response by Priming Tumor-Associated Macrophages toward M1-like Phenotype and Attenuating Tumor Hypoxia. ACS Nano.

[46] Song Y., Liu Y., Teo H.Y., Hanafi Z.B., Mei Y., Zhu Y., Chua Y.L., Lv M., Jiang Z., Liu H. (2021). Manganese enhances the antitumor function of CD8(+) T cells by inducing type I interferon production. Cell Mol. Immunol..

[47] Krezel A., Maret W. (2016). The biological inorganic chemistry of zinc ions. Arch. Biochem. Biophys..

[48] Haase H., Rink L. (2014). Multiple impacts of zinc on immune function. Metallomics.

[49] Bellomo E., Hogstrand C., Maret W. (2014). Redox and zinc signalling pathways converging on protein tyrosine phosphatases. Free Radic. Biol. Med..

[50] Truong-Tran A.Q., Carter J., Ruffin R.E., Zalewski P.D. (2001). The role of zinc in caspase activation and apoptotic cell death. Biometals.

[51] Rink L., Gabriel P. (2000). Zinc and the immune system. Proc. Nutr. Soc..

[52] Haase H., Rink L. (2014). Zinc signals and immune function. Biofactors.

[53] Wessels I., Maywald M., Rink L. (2017). Zinc as a Gatekeeper of Immune Function. Nutrients.

[54] Kim B., Lee W.W. (2021). Regulatory Role of Zinc in Immune Cell Signaling. Mol. Cells.

[55] King L.E., Frentzel J.W., Mann J.J., Fraker P.J. (2005). Chronic zinc deficiency in mice disrupted T cell lymphopoiesis and erythropoiesis while B cell lymphopoiesis and myelopoiesis were maintained. J. Am. Coll. Nutr..

[56] Beck F.W., Kaplan J., Fine N., Handschu W., Prasad A.S. (1997). Decreased expression of CD73 (ecto-5’-nucleotidase) in the CD8+ subset is associated with zinc deficiency in human patients. J. Lab. Clin. Med..

[57] Saha A.R., Hadden E.M., Hadden J.W. (1995). Zinc induces thymulin secretion from human thymic epithelial cells in vitro and augments splenocyte and thymocyte responses in vivo. Int. J. Immunopharmacol..

[58] Lin R.S., Rodriguez C., Veillette A., Lodish H.F. (1998). Zinc is essential for binding of p56(lck) to CD4 and CD8alpha. J. Biol. Chem..

[59] Yu M., Lee W.W., Tomar D., Pryshchep S., Czesnikiewicz-Guzik M., Lamar D.L., Li G., Singh K., Tian L., Weyand C.M. (2011). Regulation of T cell receptor signaling by activation-induced zinc influx. J. Exp. Med..

[60] Colomar-Carando N., Meseguer A., Company-Garrido I., Jutz S., Herrera-Fernandez V., Olvera A., Kiefer K., Brander C., Steinberger P., Vicente R. (2019). Zip6 Transporter Is an Essential Component of the Lymphocyte Activation Machinery. J. Immunol..

[61] Kaltenberg J., Plum L.M., Ober-Blobaum J.L., Honscheid A., Rink L., Haase H. (2010). Zinc signals promote IL-2-dependent proliferation of T cells. Eur. J. Immunol..

[62] Plum L.M., Brieger A., Engelhardt G., Hebel S., Nessel A., Arlt M., Kaltenberg J., Schwaneberg U., Huber M., Rink L. (2014). PTEN-inhibition by zinc ions augments interleukin-2-mediated Akt phosphorylation. Metallomics.

[63] Wellinghausen N., Driessen C., Rink L. (1996). Stimulation of human peripheral blood mononuclear cells by zinc and related cations. Cytokine.

[64] Plum L.M., Rink L., Haase H. (2010). The essential toxin: Impact of zinc on human health. Int. J. Environ. Res. Public Health.

[65] Leitzmann M.F., Stampfer M.J., Wu K., Colditz G.A., Willett W.C., Giovannucci E.L. (2003). Zinc supplement use and risk of prostate cancer. J. Natl. Cancer Inst..

[66] Singer M., Wang C., Cong L., Marjanovic N.D., Kowalczyk M.S., Zhang H., Nyman J., Sakuishi K., Kurtulus S., Gennert D. (2017). A Distinct Gene Module for Dysfunction Uncoupled from Activation in Tumor-Infiltrating T Cells. Cell.

[67] Avery J.C., Hoffmann P.R. (2018). Selenium, Selenoproteins, and Immunity. Nutrients.

[68] Ma C., Hoffmann P.R. (2021). Selenoproteins as regulators of T cell proliferation, differentiation, and metabolism. Semin. Cell Dev. Biol..

[69] Shrimali R.K., Irons R.D., Carlson B.A., Sano Y., Gladyshev V.N., Park J.M., Hatfield D.L. (2008). Selenoproteins mediate T cell immunity through an antioxidant mechanism. J. Biol. Chem..

[70] Verma S., Hoffmann F.W., Kumar M., Huang Z., Roe K., Nguyen-Wu E., Hashimoto A.S., Hoffmann P.R. (2011). Selenoprotein K knockout mice exhibit deficient calcium flux in immune cells and impaired immune responses. J. Immunol..

[71] Hoffmann P.R., Berry M.J. (2008). The influence of selenium on immune responses. Mol. Nutr. Food Res..

[72] Razaghi A., Poorebrahim M., Sarhan D., Bjornstedt M. (2021). Selenium stimulates the antitumour immunity: Insights to future research. Eur. J. Cancer.

[73] Petrie H.T., Klassen L.W., Klassen P.S., O’Dell J.R., Kay H.D. (1989). Selenium and the immune response: 2. Enhancement of murine cytotoxic T-lymphocyte and natural killer cell cytotoxicity in vivo. J. Leukoc. Biol..

[74] Xu S., Chaudhary O., Rodriguez-Morales P., Sun X., Chen D., Zappasodi R., Xu Z., Pinto A.F.M., Williams A., Schulze I. (2021). Uptake of oxidized lipids by the scavenger receptor CD36 promotes lipid peroxidation and dysfunction in CD8(+) T cells in tumors. Immunity.

[75] Carlisle A.E., Lee N., Matthew-Onabanjo A.N., Spears M.E., Park S.J., Youkana D., Doshi M.B., Peppers A., Li R., Joseph A.B. (2020). Selenium detoxification is required for cancer-cell survival. Nat. Metab..

[76] Chaigne-Delalande B., Lenardo M.J. (2014). Divalent cation signaling in immune cells. Trends Immunol..

[77] Li F.Y., Chaigne-Delalande B., Kanellopoulou C., Davis J.C., Matthews H.F., Douek D.C., Cohen J.I., Uzel G., Su H.C., Lenardo M.J. (2011). Second messenger role for Mg^2+^ revealed by human T-cell immunodeficiency. Nature.

[78] Li F.Y., Lenardo M.J., Chaigne-Delalande B. (2011). Loss of MAGT1 abrogates the Mg^2+^ flux required for T cell signaling and leads to a novel human primary immunodeficiency. Magnes. Res..

[79] Kanellopoulou C., George A.B., Masutani E., Cannons J.L., Ravell J.C., Yamamoto T.N., Smelkinson M.G., Jiang P.D., Matsuda-Lennikov M., Reilley J. (2019). Mg(2+) regulation of kinase signaling and immune function. J. Exp. Med..

[80] Ravell J.C., Chauvin S.D., He T., Lenardo M. (2020). An Update on XMEN Disease. J. Clin. Immunol..

[81] Matsuda-Lennikov M., Biancalana M., Zou J., Ravell J.C., Zheng L., Kanellopoulou C., Jiang P., Notarangelo G., Jing H., Masutani E. (2019). Magnesium transporter 1 (MAGT1) deficiency causes selective defects in N-linked glycosylation and expression of immune-response genes. J. Biol. Chem..

[82] Ravell J.C., Matsuda-Lennikov M., Chauvin S.D., Zou J., Biancalana M., Deeb S.J., Price S., Su H.C., Notarangelo G., Jiang P. (2020). Defective glycosylation and multisystem abnormalities characterize the primary immunodeficiency XMEN disease. J. Clin. Investig..

[83] Chaigne-Delalande B., Li F.Y., O’Connor G.M., Lukacs M.J., Jiang P., Zheng L., Shatzer A., Biancalana M., Pittaluga S., Matthews H.F. (2013). Mg^2+^ regulates cytotoxic functions of NK and CD8 T cells in chronic EBV infection through NKG2D. Science.

[84] Huang W.Q., Long W.Q., Mo X.F., Zhang N.Q., Luo H., Lin F.Y., Huang J., Zhang C.X. (2019). Direct and indirect associations between dietary magnesium intake and breast cancer risk. Sci. Rep..

[85] Dibaba D., Xun P., Yokota K., White E., He K. (2015). Magnesium intake and incidence of pancreatic cancer: The VITamins and Lifestyle study. Br. J. Cancer.

[86] Mahabir S., Wei Q., Barrera S.L., Dong Y.Q., Etzel C.J., Spitz M.R., Forman M.R. (2008). Dietary magnesium and DNA repair capacity as risk factors for lung cancer. Carcinogenesis.

[87] Zheng K., Yang Q., Xie L., Qiu Z., Huang Y., Lin Y., Tu L., Cui C. (2019). Overexpression of MAGT1 is associated with aggressiveness and poor prognosis of colorectal cancer. Oncol. Lett..

[88] Xie J., Cheng C.S., Zhu X.Y., Shen Y.H., Song L.B., Chen H., Chen Z., Liu L.M., Meng Z.Q. (2019). Magnesium transporter protein solute carrier family 41 member 1 suppresses human pancreatic ductal adenocarcinoma through magnesium-dependent Akt/mTOR inhibition and bax-associated mitochondrial apoptosis. Aging.

[89] Diao B., Huang X., Guo S., Yang C., Liu G., Chen Y., Wu Y. (2017). MAGT1-mediated disturbance of Mg(2+) homeostasis lead to exhausted of HBV-infected NK and CD8(+) T cells. Sci. Rep..

[90] Raza M., Chakraborty S., Choudhury M., Ghosh P.C., Nag A. (2014). Cellular iron homeostasis and therapeutic implications of iron chelators in cancer. Curr. Pharm. Biotechnol..

[91] Doherty C.P. (2007). Host-pathogen interactions: The role of iron. J. Nutr..

[92] Walker E.M., Walker S.M. (2000). Effects of iron overload on the immune system. Ann. Clin. Lab. Sci..

[93] Mims M.P., Prchal J.T. (2005). Divalent metal transporter 1. Hematology.

[94] Wang C.Y., Jenkitkasemwong S., Duarte S., Sparkman B.K., Shawki A., Mackenzie B., Knutson M.D. (2012). ZIP8 is an iron and zinc transporter whose cell-surface expression is up-regulated by cellular iron loading. J. Biol. Chem..

[95] Motamedi M., Xu L., Elahi S. (2016). Correlation of transferrin receptor (CD71) with Ki67 expression on stimulated human and mouse T cells: The kinetics of expression of T cell activation markers. J. Immunol. Methods.

[96] Zheng Y., Collins S.L., Lutz M.A., Allen A.N., Kole T.P., Zarek P.E., Powell J.D. (2007). A Role for Mammalian Target of Rapamycin in Regulating T Cell Activation versus Anergy. J. Immunol..

[97] Jabara H.H., Boyden S.E., Chou J., Ramesh N., Massaad M.J., Benson H., Bainter W., Fraulino D., Rahimov F., Sieff C. (2016). A missense mutation in TFRC, encoding transferrin receptor 1, causes combined immunodeficiency. Nat. Genet..

[98] Yarosz E.L., Ye C., Kumar A., Black C., Choi E.-K., Seo Y.-A., Chang C.-H. (2020). Cutting Edge: Activation-Induced Iron Flux Controls CD4 T Cell Proliferation by Promoting Proper IL-2R Signaling and Mitochondrial Function. J. Immunol..

[99] Wang Z., Yin W., Zhu L., Li J., Yao Y., Chen F., Sun M., Zhang J., Shen N., Song Y. (2018). Iron Drives T Helper Cell Pathogenicity by Promoting RNA-Binding Protein PCBP1-Mediated Proinflammatory Cytokine Production. Immunity.

[100] Liu Y., Duan C., Dai R., Zeng Y. (2021). Ferroptosis-mediated Crosstalk in the Tumor Microenvironment Implicated in Cancer Progression and Therapy. Front. Cell Dev. Biol..

[101] Shaw J., Chakraborty A., Nag A., Chattopadyay A., Dasgupta A.K., Bhattacharyya M. (2017). Intracellular iron overload leading to DNA damage of lymphocytes and immune dysfunction in thalassemia major patients. Eur. J. Haematol..

[102] Brown R.A.M., Richardson K.L., Kabir T.D., Trinder D., Ganss R., Leedman P.J. (2020). Altered Iron Metabolism and Impact in Cancer Biology, Metastasis, and Immunology. Front. Oncol..

[103] Yu Y., Xie Y., Cao L., Yang L., Yang M., Lotze M.T., Zeh H.J., Kang R., Tang D. (2015). The ferroptosis inducer erastin enhances sensitivity of acute myeloid leukemia cells to chemotherapeutic agents. Mol. Cell Oncol..

[104] Waldman A.D., Fritz J.M., Lenardo M.J. (2020). A guide to cancer immunotherapy: From T cell basic science to clinical practice. Nat. Rev. Immunol..

[105] Feske S., Skolnik E.Y., Prakriya M. (2012). Ion channels and transporters in lymphocyte function and immunity. Nat. Rev. Immunol..

[106] Huang H., Zhou P., Wei J., Long L., Shi H., Dhungana Y., Chapman N.M., Fu G., Saravia J., Raynor J.L. (2021). In vivo CRISPR screening reveals nutrient signaling processes underpinning CD8(+) T cell fate decisions. Cell.

[107] Canale F.P., Basso C., Antonini G., Perotti M., Li N., Sokolovska A., Neumann J., James M.J., Geiger S., Jin W. (2021). Metabolic modulation of tumours with engineered bacteria for immunotherapy. Nature.

[108] Reinfeld B.I., Madden M.Z., Wolf M.M., Chytil A., Bader J.E., Patterson A.R., Sugiura A., Cohen A.S., Ali A., Do B.T. (2021). Cell-programmed nutrient partitioning in the tumour microenvironment. Nature.

